# Viral Deregulation of Apoptotic Pathways and Its Correlation With Adverse Pregnancy Outcomes

**DOI:** 10.7759/cureus.68095

**Published:** 2024-08-29

**Authors:** Stylianos Tologkos, Vasiliki Papadatou, Vasiliki Lampropoulou, Olga Pagonopoulou, Christina Angelika Alexiadi, Triantafyllos Alexiadis, Gregory Trypsianis, Soultana Meditskou, Maria Lambropoulou

**Affiliations:** 1 Histology-Embryology, Democritus University of Thrace, Alexandroupolis, GRC; 2 Department of Physiology, Democritus University of Thrace, Alexandroupolis, GRC; 3 Laboratory of Histology-Embryology, School of Medicine, Democritus University of Thrace, Alexandroupolis, GRC; 4 Biostatistics, School of Medicine, Democritus University of Thrace, Alexandroupolis, GRC; 5 Laboratory of Histology-Embryology, School of Medicine, Faculty of Health Sciences, Aristotle University of Thessaloniki, Thessaloniki, GRC

**Keywords:** virus, pregnancy, spontaneous abortion, ebv, b19, apoptosis

## Abstract

Objective: Our objective was to correlate parvovirus-B19 and Epstein-Barr virus (EBV) infections with apoptotic biomarker levels in tissues from placentas from spontaneous abortions and cases of elective termination of pregnancy. We also explored if viral presence could cause spontaneous abortions by trying to associate the levels of pro-apoptotic markers with adverse pregnancy outcomes.

Materials and methods: We used 194 placental samples, of which 152 came from spontaneous abortions and were the study group and 42 controls came from cases of elective pregnancy termination. Hematoxylin and eosin (H&E) staining was performed to investigate morphological changes in the tissues, and then indirect immunohistochemistry to evaluate the expression of B19, EBV, M30, terminal deoxynucleotidyl transferase assay (TUNEL), and nuclear factor kappa B (NF-kB). Statistical analysis was performed using SPSS v. 19.0 (IBM).

Results: Higher levels of apoptosis were observed in the spontaneous abortion group (p<0.001) with statistical significance and their presence was also correlated with statistical significance with viral infection (p<0.001). Also, viral infections were observed only in cases of spontaneous abortion. When simple and multivariate logistic regression was performed we confirmed that viral presence remained an independent prognostic factor for high expression of all apoptotic biomarkers with statistical significance (p<0.001).

Conclusions: Our results indicate that viral presence can lead to deregulation of apoptotic pathways within the maternal-fetal environment and thus work as a trigger event for spontaneous abortions.

## Introduction

The Epstein-Barr virus (EBV), belonging to the herpes family and known as human herpesvirus 4, is a common viral infection affecting humans worldwide [1]. It is one of the most prevalent viruses in humans, with most people getting infected at some point in their lives [2]. The virus stays in the human body for the remainder of their life and its chronic reactivation can lead to the onset of many diseases later in life [3]. EBV is primarily transmitted by saliva, although it can also be passed on through sperm or blood [2]. Infectious mononucleosis is one of the most well-known conditions linked to EBV [4]. The risks associated with EBV infection during pregnancy are not fully described and there is still uncertainty about whether EBV infection during pregnancy increases the risk of abortion or prematurity [5]. Parvovirus B19 (B19V) is a member of the broad Parvoviridae family and can cause various pathologies [6]. B19 affects approximately 1-5% of women during their pregnancy, most of whom go on to have a positive pregnancy outcome. The infection however in some cases can lead to spontaneous miscarriage, nonimmune hydrops, and congenital anomalies [7]. During the second trimester, the vertical transmission of the virus to the fetus can have a fatal outcome to the embryo, with intrauterine death in the third trimester being just as significant [8,9]. The apoptosis pathway is observed during the process of placenta formation, and the presence of apoptotic biomarkers can be associated with specific pregnancy disorders. In the normal course of pregnancy, trophoblast apoptosis exhibits a gradual increase over time [10]. Apoptosis, a natural process of programmed cell death, is a normal part of trophoblast turnover in both villous and extravillous trophoblast compartments. However, when dysregulated, excessive apoptosis in these cells can contribute to miscarriages. The correct balance of apoptosis is crucial for healthy placental development and function. Research highlights the participation of apoptosis in the cytotrophoblast and syncytiotrophoblast stages within the placenta. Dysregulated apoptosis can disrupt the delicate maternal-fetal interface and potentially trigger inflammatory reactions by the maternal host, contributing to pregnancy complications and miscarriages [11-13].

## Materials and methods

Samples

A total of 194 paraffin tissue sections with placental tissue were obtained from the archive of the Laboratory of Histology & Embryology of Democritus University of Thrace. These were divided into two groups. The study group consisted of 152 samples from spontaneous abortions and the control group comprised 42 samples of intentional termination of pregnancy. The approval number for research on our archive material by the local Bioethics and Human Investigations Committee is Ref Num. 45/27/HMB/16.11.2009, which ruled that it was in conformity with the local legislation, as well as with the Code of Ethics of the World Medical Association (Declaration of Helsinki).

Ethical considerations

The study was conducted according to the criteria set by the 1964 Helsinki Declaration and its later amendments.

Staining

According to the standard histological protocol, all samples were fixed in 10% buffered formalin and embedded in paraffin. 3-μm hematoxylin-eosin-stained sections were histopathologically examined. Serial sections from each case were deparaffinized, rehydrated, and treated with 0.3% H_2_O_2_. Immunohistochemical staining was performed using the following antibodies: anti-B19 (Dako, Glostrup, Denmark -Rabbit Polyclonal 1:200 dilution, specific for the VP2 protein), anti-EBV (Dako, Glostrup, Denmark -Rabbit Polyclonal 1:150 dilution, specific for the LMP protein), and M30 (Alexis Biochemistry 1:80 dilution, specific for an epitope of creatine-18) and was visualized by the peroxidase method (Envision System, DAKO, Carpinteria, CA, USA) using EnVisionTM FLEX diaminobenzidine (DAB) chromogen. The Terminal deoxynucleotidyl-transferase mediated dUTP Nick End Labeling (TUNEL) method was used for the identification of apoptotic cells. The immunochemical protocol for this method is essentially the same, with the exception that instead of adding an antibody to an antigen, a solution containing Terminal Deoxynucleotide I transferase (Tdt) mediated dUTP Nick End Labeling with 450 μl Label Solution and 50 μl Enzyme Solution was added and incubated overnight.

Immunohistochemical evaluation

All samples were studied using a Nikon Eclipse 50i microscope with an integrated camera Nikon Digital Sight DS-L1 (Nikon Corporation, Tokyo, Japan). From each histological section, 5 high-power fields were randomly chosen. Evaluation of antibody expression was performed using a semi-quantitative system, where no expression was valued as 0 (<10% stained cells), low expression (10-30%) as 1 medium (30-70%), and high expression (>70%) as 2 and 3, respectively.

Statistical analysis

Statistical analysis of our data was performed using the package SPSS v. 19.0 (IBM). Quantitative variables were described as median values with ±1 standard deviation (SD). On the other hand, qualitative parameters of the patients were shown as absolute and relative frequencies (%). We performed the chi-squared test to investigate the correlation between the expression of the tested biomarkers with patient clinical and histological characteristics. Evaluation of the possibility for positive expression of B19, EBV, M30, TUNEL, and NF-kB was performed through the calculation of odds ratio (OR) and the corresponding 95% confidence intervals (CI) through simple logistic regression models. Multiple logistic regression was then performed in order to investigate independent prognostic factors for positive expression of the biomarkers. All statistical tests were bilateral, and the results were considered statistically significant for p<0.05.

## Results

**Figure 1:**
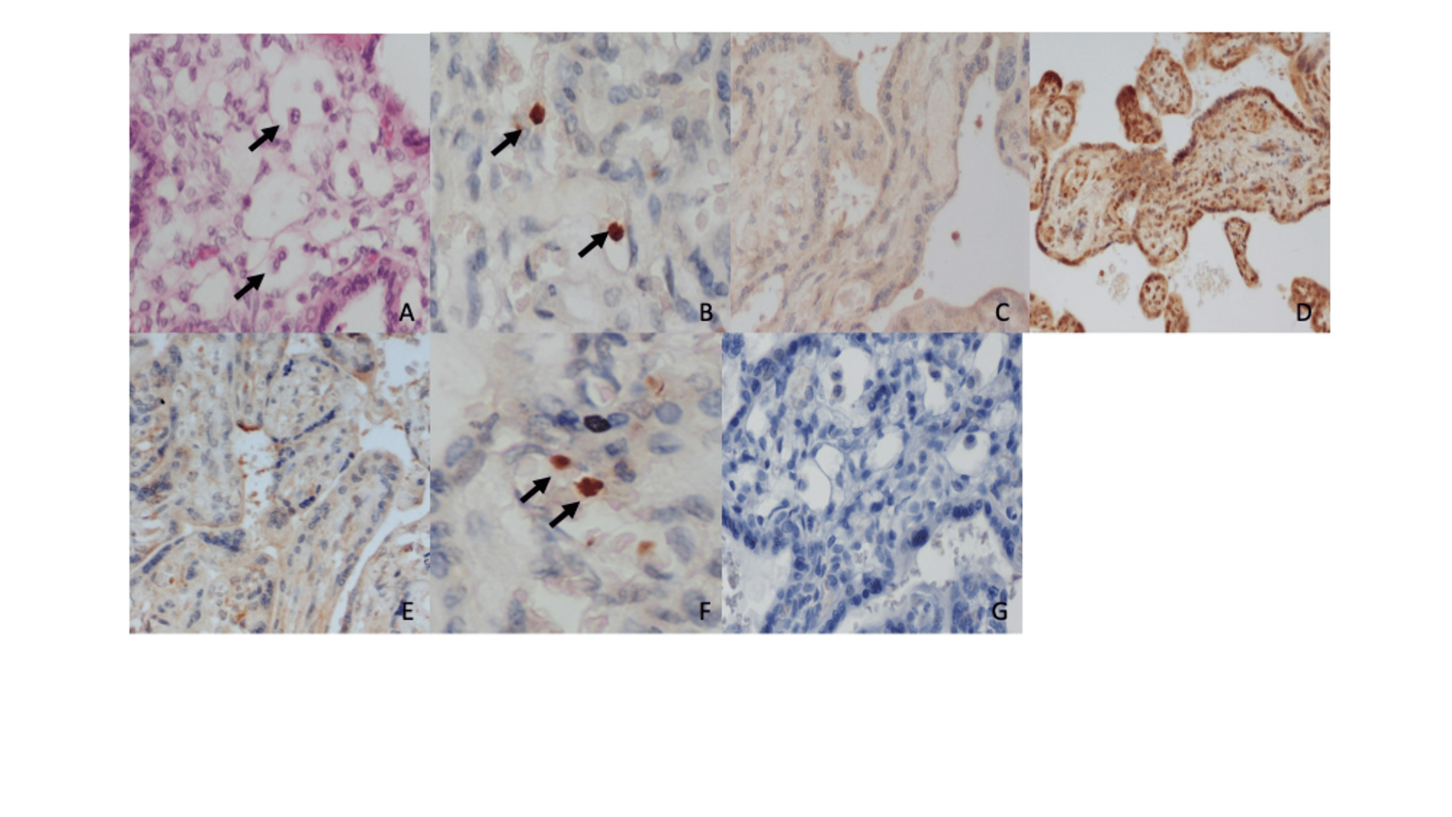
Immunohistochemistry and hematoxylin & eosin staining results Α) Hematoxylin and eosin staining in virus-positive sample (×200). Inclusion bodies are shown; B) Positive EBV expression in placentas from spontaneous abortion cases shown by arrows (×400); C) Medium expression of TUNEL in placental tissue from a spontaneous abortion case (×200); D) High expression of NF-kB in placental tissue from a spontaneous abortion case (×200); E) Medium expression of M30 in placental tissue from a spontaneous abortion case (×200); F) Positive B19 expression in placentas from spontaneous abortion cases shown by arrows (×400); G) Negative marker for immunohistochemistry staining (×200) TUNEL: Terminal deoxynucleotidyl-transferase-mediated dUTP Nick End Labeling; NF-kB: nuclear factor kappa B; EBV: Epstein-Barr virus

Immunohistochemistry and hematoxylin & eosin staining are shown in Figure 1.

The clinical and demographic characteristics of patients are presented in Table 1.

**Table 1:** Comparison of the demographic and clinical characteristics between the two sample groups. EBV: Epstein-Barr virus

Groups			
	Control	Study	P-Value
Age			0.908
<25	13 (31.0)	44 (28.9)	-
26-34	24 (49.0)	56 (43.8)	-
≥35	11 (22.4)	40 (31.3)	-
Trimester			<0.001
1^st^	30 (71.4)	51 (33.6)	-
2^nd^	12 (28.6)	58 (3 28.2)	-
3^rd^	0 (0.0)	43 (28.3)	-
Sex			0.203
Male	24 (57.1)	70 (46.1)	-
Female	18 (42.9)	82 (53.9)	-
Β19			0.080
Negative	42 (100.0)	142 (93.4)	-
Positive	0 (0.0)	10 (6.6)	-
EBV			0.106
Negative	42 (100.0)	143 (94.1)	-
Positive	0 (0.0)	9 (5.9)	-

Expression of apoptotic biomarkers is shown in Table 2. 

**Table 2:** Comparison of the expression of apoptotic markers in the two sample groups. TUNEL: Terminal deoxynucleotidyl-transferase-mediated dUTP Nick End Labeling; NF-κΒ: nuclear factor kappa B

	Groups		
	Control	Study	P-Value
TUNEL			<0.001
0	29 (69.0)	5 (3.3)	-
1	12 (28.6)	61 (40.1)	-
2	1 (2.4)	72 (47.4)	-
3	0 (0.0)	16 (10.5)	-
M-30			<0.001
0	32 (76.2)	6 (3.9)	-
1	9 (21.4)	58 (38.2)	-
2	1 (2.4)	60 (46.9)	-
3	0 (0.0)	29 (22.7)	-
NF-κΒ			<0.001
0	33 (78.6)	5 (3.3)	-
1	9 (21.4)	66 (43.4)	-
2	0 (0.0)	62 (40.8)	-
3	0 (0.0)	19 (12.5)	-

Terminal deoxynucleotidyl-transferase-mediated dUTP Nick End Labeling (TUNEL) expression in the study group compared to patient characteristics

The correlation of TUNEL expression with patient characteristics is given in Table 3. The use of a chi-squared test showed that stronger TUNEL expression was statistically significantly associated with older maternal age (p=0.023), as well as with the presence of viral factor (p<0.001). In contrast, there was no statistically significant correlation of TUNEL expression with child sex (p=0.140) or trimester of gestation (p=0.247). The use of the chi-squared test showed that high levels of TUNEL were more commonly found in patients positive for EBV with 55.6% of those patients having high TUNEL expression compared to 3.5% of the negative ones (p<0.001) (Table 3). As for the B19 virus, the chi-squared test indicated that high levels of TUNEL were more common in positive for the virus patients, as 50% of those patients had high TUNEL expression compared to 3.5% for patients that were negative for viral presence(p<0.001). Using simple logistic regression, it was shown that positive TUNEL expression was 34.50 times more frequent in patients positive for EBV presence (OR=34.50, 95% CI=7.04-169.00, p<0.001) compared to patients negative for the virus. (Table 3). Using the same method for the B19 virus, the results indicated that TUNEL was 27.40 times more frequent in patients positive for B19 (OR=27.40, 95% CI=5.95-126.21, p<0.001) than in patients that the virus was not found (Table 3). Multiple logistic regression revealed that the presence of viruses (p<0.001) was still an independent predictor of high (≥30%) TUNEL expression (Table 3). 

**Table 3:** Levels of TUNEL expression in a study group in comparison with clinical parameters. High TUNEL expression is associated with the demographic and clinical characteristics of the study group. TUNEL: Terminal deoxynucleotidyl-transferase-mediated dUTP Nick End Labeling; EBV: Epstein-Barr virus

-	TUNEL expression				-	-
--	Negative	Low	Medium	High	P-Value	-
Age					0.023	-
<25	4 (9.1)	21 (47.7)	18 (40.9)	1 (2.3)	-	-
26-34	1 (1.6)	27 (42.9)	32 (50.8)	3 (4.8)	-	-
≥35	0 (0.0)	13 (28.9)	26 (57.8)	6 (1.3)	-	-
Trimester					0.247	-
1^st^	4 (7.8)	23 (45.1)	20 (39.2)	4 (7.8)	-	-
2^nd^	0 (0.0)	22 (37.9)	32 (55.2)	4 (6.9)	-	-
3^rd^	1 (2.3)	16 (37.2)	24 (55.8)	2 (4.7)	-	-
Sex					0.140	-
Male	4 (5.7)	28 (40.0)	31 (44.3)	7 (10.0)	-	-
Female	1 (1.2)	33 (40.2)	45 (54.9)	3 (3.7)	-	-
Β19					<0.001	-
Negative	5 (3.5)	61 (43.0)	71 (50.0)	5 (3.5)	-	-
Positive	0 (0.0)	0 (0.0)	5 (50.0)	5 (50.0)	-	-
EBV					<0.001	-
Negative	5 (3.5)	61 (42.7)	72 (50.3)	5 (3.5)	-	-
Positive	0 (0.0)	0 (0.0)	4 (44.4)	5 (55.6)	-	-
Presence of high TUNEL expression						-
-	No (%)	P-Value	cOR (95% CI)	P-Value	aOR (95% CI)	P-Value
Age	-	0.082	-	-	-	-
<25	1 (2.3)	-	Ref.	-	Ref.	-
26-34	3 (4.8)	-	2.15 (0.22-21.38)	0.514	-	-
≥35	6 (13.3)	-	6.62 (0.76-57.42)	0.087	-	-
Trimester	-	0.419	-	-	-	-
1^st^	4 (7.8)	-	Ref.	-	-	
2^nd^	4 (6.9)	-	0.870 (0.21-3.67)	0.850	-	-
3^rd^	2 (4.7)	-	0.573 (0.10-3.29)	0.533	-	-
Sex		0.345	-	-		-
Male	7 (10.0)	-	Ref.	-	-	-
Female	3 (3.7)	-	0.34 (0.09-1.38)	0.131	-	-
Β19		<0.001		-	-	-
Negative	5 (3.5)	-	Ref.	-	Ref.	-
Positive	5 (50.0)	-	27.40 (5.95-126.21)	<0.001	27.40 (5.95-126.21)	<0.001
EBV		<0.001	-	-	-	-
Negative	5 (3.5)	-	Ref.	-	Ref.	-
Positive	5 (55.6)	-	34.50 (7.04-169.00)	<0.001	34.50 (7.04-169.00)	<0.001

M30 expression in the study group compared to patient characteristics

Table 4 shows the relationship between M30 expression and patient characteristics. Stronger M30 expression showed a statistically significant correlation with older maternal age (p=0.005) and the presence of viral factors (p<0.001), according to the chi-squared test. On the other hand, M30 expression did not significantly correlate with either the trimester of gestation (p=0.090) or child sex (p=0.762). The chi-squared test revealed that high M30 expression was statistically significantly more common in patients positive for EBV viral presence, with 88.9% of positive patients having high expression compared to 5.6% of those negative for viral presence (p<0.001), and in women of older age (>35 years) compared to younger ones (25 years) (p=0.047) (Table 3). The same findings were observed for the B19 virus, with high M30 expression being statistically significantly more common in patients positive for B19; in patients positive for B19 viral presence, high expression was found in 70% of cases compared to 6.3% of cases negative for viral presence (p<0.001), and in women older than 35 years old compared to younger women (p=0.047) (Table 3). Using simple logistic regression, positive M30 expression was found to be 135 times more frequent in patients positive for EBV presence (OR=135, 95% CI=14.99-1215.69, p<0.001) compared to patients in the absence of the virus (Table 3). The same method was used to determine the prevalence of the B19 virus, and the results showed that individuals with the virus were 34.48 times more likely to have positive M30 expression than those who did not have it (OR=34.48, 95% CI=7.61-156.34, p<0.001). The results of multiple logistic regression demonstrated that the presence of any virus (p<0.001) continued to be an independent indicator of high (≥30% ) M30 expression. Particularly, individuals with EBV presence had positive M30 expression 135 times more than those not infected with EBV (adjusted OR=135, 95% CI=14.99-1215.69, p<0.001) (Table 4) and those infected with the B19 virus had positive M30 expression 32.84 times more than the negative ones (adjusted OR=32.84, 95% CI=6.83-157.82, p<0.001).

**Table 4:** Levels of M30 expression in study group in comparison with clinical parameters. High M30 expression is associated with the demographic and clinical characteristics of the study group. EBV: Epstein-Barr virus; NF-kB: nuclear factor kappa B

-	M30 expression				-	-
-	Negative	Low	Medium	High	P-Value	-
Age					0.005	-
<25	2 (4.5)	22 (50.0)	17 (38.6)	3 (6.8)	-	-
26-34	4 (6.3)	28 (44.4)	27 (42.9)	4 (6.3)	-	-
≥35	0 (0.0)	8 (17.8)	28 (62.2)	9 (20.0)	-	-
Trimester					0.090	-
1^st^	5 (9.8)	23 (45.1)	19 (37.3)	4 (7.8)	-	-
2^nd^	0 (0.0)	22 (37.9)	30 (51.7)	6 (10.3)	-	-
3^rd^	1 (2.3)	13 (30.2)	23 (53.5)	6 (14.0)	-	-
Sex					0.762	-
Male	3 (4.3)	25 (35.7)	36 (51.4)	6 (8.6)	-	-
Female	3 (3.7)	33 (40.2)	36 (43.9)	10 (12.2)	-	-
Β19					<0.001	-
Negative	6 (4.2)	58 (40.8)	69 (48.6)	9 (6.3)	-	-
Positive	0 (0.0)	0 (0.0)	3 (30.0)	7 (70.0)	-	-
EBV					<0.001	-
Negative	6 (4.2)	58 (40.6)	71 (49.7)	8 (5.6)	-	-
Positive	0 (0.0)	0 (0.0)	1 (11.1)	8 (88.9)	-	-
Presence of high M30 expression						-
-	No (%)	P-Value	cOR (95% CI)	P-Value	aOR (95% CI)	P-Value
Age	-	0.047	-	-	-	-
<25	3 (6.8)	-	Ref.	-	Ref.	-
26-34	4 (6.3)	-	0.93 (0.20-4.36)	0.923	-	-
≥35	9 (20.0)	-	3.42 (0.86-13.60)	0.081	-	-
Trimester		0.629	-	-	-	-
1^st^	4 (7.8)	-	Ref.	-	-	-
2^nd^	6 (10.3)	-	1.36 (0.36-5.10)	0.653	-	-
3^rd^	6 (14.0)	-	1.91 (0.50-7.25)	0.344	-	-
Sex	-	0.486	-	-	-	-
Male	6 (8.6)		Ref.		-	-
Female	10 (12.2)		1.48 (0.51-4.31)	0.470	-	-
Β19	-	<0.001	-	-	-	-
Negative	10 (7.0)	-	Ref.	-	Ref.	-
Positive	6 (60.0)	-	34.48 (7.61-156.34)	<0.001	32.84 (6.83-157.82)	<0.001
EBV	-	<0.001	-	-	-	-
Negative	8 (5.6)	-	Ref.	-	Ref.	-
Positive	8 (88.9)	-	135 (14.99-1215.69)	<0.001	135.00 (14.99-1215.68)	<0.001

Nuclear factor kappa B (NF-kB) expression in the study group compared to patient characteristics

The correlation between NF-kB expression and patient characteristics is shown in Table 5.

**Table 5:** Levels of NF-kB expression in study group in comparison with clinical parameters. High NF-kB expression is associated with the demographic and clinical characteristics of the study group. NF-kB: nuclear factor kappa B; EBV: Epstein-Barr virus

-	NF-κΒ expression				-	-
-	Negative	Low	Medium	High	P-Value	-
Age					0.019	-
<25	3 (6.8)	25 (56.8)	14 (31.8)	2 (4.5)	-	-
26-34	2 (3.2)	26 (41.3)	29 (46.0)	6 (9.5)	-	-
≥35	0 (0.0)	15 (33.3)	19 (42.2)	11 (24.4)	-	-
Trimester					0.011	-
1^st^	4 (7.8)	28 (54.9)	14 (27.5)	5 (9.8)	-	-
2^nd^	1 (1.7)	17 (29.3)	33 (56.9)	7 (12.1)	-	-
3^rd^	0 (0.0)	21 (48.8)	15 (34.9)	7 (16.3)	-	-
Sex					0.933	-
Male	3 (4.3)	30 (42.9)	28 (40.0)	9 (12.9)	-	-
Female	3 (2.4)	36 (43.9)	34 (41.5)	10 (12.2)	-	-
Β19					<0.001	-
Negative	5 (3.5)	66 (46.5)	61 (43.0)	19 (7.0)	-	-
Positive	0 (0.0)	0 (0.0)	1 (10.0)	9 (90)	-	-
EBV					<0.001	-
Negative	5 (3.5)	66 (46.2)	61 (42.7)	11 (7.7)	-	-
Positive	0 (0.0)	0 (0.0)	1 (11.1)	8 (88.9)	-	-
Presence of high NF-kB expression						-
	No (%)	P-Value	cOR (95% CI)	P-Value	aOR (95% CI)	P-Value
Age	-	0.012	-	-	-	-
<25	2 (4.5)	-	Ref.	-	Ref.	-
26-34	6 (9.5)	-	2.21 (0.43-11.50)	0.346	-	-
≥35	11 (24.4)		6.79 (1.41-32.76)	0.017	2.87 (0.87-9.48)	0.083
Trimester	-	0.419	-	-	-	-
1^st^	5 (9.8)	-	Ref.	-	-	-
2^nd^	7 (12.1)	-	1.26 (0.38-4.26)	0.707	-	-
3^rd^	7 (16.3)	-	1.79 (0.52-6.11)	0.353	-	-
Sex	-	0.902	-	-	-	-
Male	9 (12.9)		Ref.		-	-
Female	10 (12.2)		0.94 (0.36-2.47)	0.902	-	-
Β19	-	<0.001	-	-	-	-
Negative	10 (7.0)	-	Ref.	-	Ref.	-
Positive	9 (90.0)	-	118.80 (13.65-1033.94)	<0.001	190.94 (18.24-1999.17)	<0.001
EBV	-	<0.001	-	-	-	-
Negative	11 (7.7)	-	Ref.	-	Ref.	-
Positive	8 (88.9)	-	96.00 (10.98-839.07)	<0.001	77.53 (8.60-698.75)	<0.001

The chi-squared test revealed that the presence of viral factor (p<0.001), a longer gestational trimester (p=0.011), and an older maternal age (p=0.019) were all statistically significantly linked with greater NF-kB expression. In contrast, there was no statistically significant correlation between the child's sex and NF-kB expression (p=0.933). The use of a chi-squared test revealed that high expression of NF-kB was statistically significantly more common in patients who tested positive for EBV presence, with 88.9% of positive patients showing high expression compared to 7.7% of negative patients (p<0.001), as well as in women of the oldest age (>35 years) compared to those of the youngest age (25 years) (p<0.012) (Table 5). Results for the B19 virus were similar, with patients who were infected with the B19 virus having higher NF-kB expression (90%) than the negative ones (7.0%) (p=0.002). Additionally, women aged over 35 demonstrated higher NF-kB expression compared to those aged 25 or younger (p<0.001). Moreover, older women exhibited elevated NF-kB levels (p=0.012). These findings are presented also in Table 5. Using simple logistic regression, positive NF-kB expression was found to be 6.79 times more prevalent in women over 35 years old compared to those under 25 years old (OR=6.79, 95% CI=1.41-32.76, p=0.017) and 96.00 times more common in patients positive for EBV (OR=96.00, 95% CI=10.98-839.07, p<0.001). For B19 NF-kB was indicated to be 118.80 times more common in positive patients (OR=118.70, 95% CI=13.65-1033.94, p<0.001) (Table 5). Increased maternal age (p=0.083) and the presence of EBV (p<0.001) were found to be independent predictors of high (≥30%) NF-kB expression by multiple logistic regression. Compared to patients without the virus, positive NF-kB expression was 2.87 times greater in women over 35 (adjusted OR=2.87, 95% CI=0.87-9.48, p=0.083) and 77.53 times higher in patients with viral presence (adjusted OR=77.53, 95% CI=8.60-698.75, p<0.001). B19 virus was detected using the same approach, which revealed that increasing maternal age (p=0.006) and B19 virus presence (p<0.001) remained independent predictors of high (≥30%) NF-kB expression. Particularly, compared to patients in whom the virus was absent, positive NF-kB expression was 190.94 times greater in patients with viral presence (adjusted OR=190.94, 95% CI=18.24-1999.17, p<0.001) and 7.08 times higher in older women patients (adjusted OR=7.08, 95% CI=1.76-28.40, p=0.006) (Table 5). 

## Discussion

Viral infections can lead to multiple complications during pregnancy with the potential fetal consequences being significant. In pregnant women, parvovirus B19 infection is typically asymptomatic, but in about 3% of infected women, it can lead to a variety of problems, including miscarriage, severe fetal anemia, nonimmune hydrops fetalis, and even fetal death [14]. EBV virus has a well-established ability to reactivate during pregnancy. Significant EBV reactivation during pregnancy may influence pregnancy duration. This shows that EBV reactivation and pregnancy outcomes can be linked [15]. In the present study, we aimed to correlate viral presence with higher levels of apoptotic markers as well as with pregnancy outcomes. Initially, we conducted an analysis of apoptosis markers, specifically M30, TUNEL, and NF-kB expression, within two distinct sample groups. Subsequently, we kept exploring a potential correlation between the presence of viruses and the expression levels of these biomarkers in the group experiencing spontaneous abortions. The objective was to clarify any potential influence of viruses in apoptotic pathways in the placental environment. Numerous investigations have looked into the expression of various apoptotic markers in the placenta and any potential links to unfavorable pregnancy outcomes up until this point [16-18]. In this present study, we conducted a comparative analysis of M30, TUNEL, and NF-kB apoptotic markers in placental samples collected from all three trimesters of pregnancy. Our findings reveal a consistent and statistically significant connection between the presence of the two viruses and the apoptotic biomarkers, and the spontaneous abortions or intrauterine deaths. Viral infections appear to activate the apoptotic pathway in placental tissue, a process that results in pregnancy complications. These results are consistent with other studies that suggest the presence of viruses during pregnancy may lead to adverse pregnancy outcomes [18-22]. The presence of apoptotic markers in placental tissues during pregnancy in virus-positive women is associated with several complications of pregnancy. However, it is crucial to emphasize that further research is demanded to elucidate the precise role of these markers in the progression of pregnancy and their impact on the well-being of both the mother and the developing fetus. Moreover, this study didn't evolve any pathway-specific apoptotic markers, because the aim was to correlate apoptosis in general with viral infections and miscarriages. Further research needs to be performed in order to elucidate which specific apoptotic pathway is activated in these conditions. It is important to acknowledge that our current understanding of this complex relationship is incomplete, and ongoing investigations are essential to provide a broad insight into the mechanisms and implications of these apoptotic markers in the context of pregnancy.

## Conclusions

In this study, we have demonstrated that viral infections, such as parvovirus B19 and EBV, are associated with the deregulation of apoptotic pathways in placental tissues. This deregulation can be measured through the elevated levels of apoptotic biomarkers such as M30, TUNEL, and NF-kB. Our findings underscore the significant role that viral infections can play in triggering adverse pregnancy outcomes, including spontaneous abortions. The presence of these viruses in the maternal-fetal environment appears to act as a catalyst for apoptosis, thereby contributing to pregnancy complications. These results align with previous research suggesting that viral presence during pregnancy may be a critical factor in adverse outcomes. However, further research is needed to fully elucidate the complex interactions between viral infections, apoptotic pathways, and pregnancy outcomes to better understand the mechanisms at play and to potentially use this information in order to take better more informed clinical decisions for the management of pregnancies affected by such viral infections.
